# Psychometric validation of the spanish SDM-20: an occupational health screening tool for assessing workplace mobbing

**DOI:** 10.13075/ijomeh.1896.02836

**Published:** 2026

**Authors:** Katarzyna Durniat, Pedro Antonio Díaz Fúnez, Marta Kowal, Miguel Ángel Mañas Rodríguez

**Affiliations:** ^1^ University of Wrocław, Institute of Psychology, Faculty of Historical and Pedagogical Sciences, Wrocław, Poland; ^2^ University of Almeria, Research Team in Psychology of Work, Organizations and Human Resources (IPTORA), Faculty of Psychology, Almeria, Spain

**Keywords:** occupational health, workplace bullying, health surveillance, psychometric validation, psychological harm, mobbing

## Abstract

**Objectives:**

The aim of this study was to psychometrically validate the Spanish version of the Polish SDM-20 questionnaire (*Skala Doświadczania Mobbingu*) as a screening instrument for assessing workplace mobbing in occupational health settings.

**Material and Methods:**

A cross-sectional online survey was conducted among 753 Spanish employees (65.5% women, age: 18–66 years). The factorial structure of the SDM-20 was examined using confirmatory factor analysis (CFA). Internal reliability was assessed using Cronbach's α and McDonald's ω coefficients. Convergent and divergent validity were evaluated through correlations with workplace bullying (*Negative Acts Questionnaire – Revised* – NAQ-R), burnout (*Maslach Burnout Inventory – General Survey –* MBI-GS9), and organizational climate measures.

**Results:**

Confirmatory factor analysis supported the original 3-factor model, encompassing person-related mobbing, work-related mobbing, and health-related harm, and demonstrated satisfactory model fit. All subscales showed high internal reliability. Strong positive correlations with workplace bullying and burnout measures supported convergent validity, whereas negative associations with organizational climate indicators provided evidence of divergent validity. The results confirm the structural stability and construct validity of the Spanish SDM-20.

**Conclusions:**

The Spanish version of the SDM-20 is a reliable and valid instrument for screening workplace mobbing in occupational health contexts. By integrating behavioral exposure with a psychometrically assessed harm component, the instrument enables differentiation between exposure and clinically relevant victimization. Its brevity and robust psychometric properties support its application in occupational health surveillance and early risk detection.

## Highlights

- The study validated the Spanish version of the SDM-20 mobbing questionnaire.
- The Spanish SDM-20 demonstrated strong reliability and validity.
- The instrument integrates behavioural exposure with psychological harm.
- The SDM-20 distinguishes between mobbing targets and victims.
- The validated tool supports health screening and early risk detection.

## INTRODUCTION

Contemporary occupational medicine and organisational psychology face a growing challenge in the accurate assessment and monitoring of workplace mobbing, referred to in Anglo-Saxon literature as workplace bullying. Since the 1990s, this phenomenon has evolved from being perceived primarily as an interpersonal or organisational problem into a critical issue for public health and occupational safety [1,2]. Workplace mobbing is commonly defined as repetitive and prolonged exposure of employees to negative and unwanted behaviours that have a detrimental impact on mental health, occupational functioning, and overall well-being [3]. Epidemiological research indicates that 10–20% of employees report exposure to workplace mobbing, although prevalence estimates vary depending on methodological approaches and measurement strategies [4,5].

A substantial and methodologically robust body of empirical research has consistently demonstrated that exposure to mobbing behaviours is associated with a wide spectrum of adverse mental and somatic health outcomes [3,6]. These include psychological distress, anxiety, depressive symptoms [7], emotional exhaustion and sleep disturbances [8], as well as post-traumatic stress symptoms and increased risk of sickness absence, work disability, and turnover [2,9,10]. Importantly, the impact of mobbing extends beyond mental health to encompass psychosomatic complaints such as musculoskeletal pain, gastrointestinal disturbances, and cardiovascular problems, reflecting chronic stress-related physiological dysregulation [7–10]. Longitudinal evidence further indicates that these effects may be persistent, contributing to long-term deterioration in mental health and functioning [7,10]. Collectively, this evidence supports the conceptualisation of mobbing as a chronic interpersonal stressor with clinically significant consequences for both mental and physical health [3,6].

The growing evidence on workplace mobbing is increasingly reflected in regulatory approaches to psychosocial risk management in occupational health. In Spain, mobbing is recognised as an occupational risk under the Occupational Risk Prevention Act [11], which obliges employers to assess and manage psychosocial hazards affecting workers' health. National technical guidelines issued by the Spanish National Institute for Safety and Health at Work (INSST), particularly NTP 854, define mobbing as systematic and prolonged psychological violence posing a threat to health [12]. From this perspective, the availability of valid and clinically sensitive assessment instruments is essential for occupational health surveillance, prevention, and medico-legal decision-making [13].

Despite this growing recognition, significant methodological challenges persist in the assessment of workplace mobbing. A key limitation lies in its inadequate operationalisation, particularly with respect to health-related harm [14–16]. Most studies rely either on self-labelling approaches, which are vulnerable to cognitive biases, or on behavioural experience methods focusing on the frequency of negative acts while largely neglecting subjective appraisal and health impairment [4,5]. Although combined approaches have been proposed, they remain insufficient to capture the interactional and clinically relevant nature of mobbing processes [17,18]. This limitation is also evident in the Spanish context, where widely used instruments – primarily adaptations of the *Negative Acts Questionnaire – Revised* (NAQ-R) – focus predominantly on behavioural exposure and offer limited capacity to distinguish between exposure and health-impairing victimisation [13,19]. This highlights the need for measurement approaches that integrate behavioural exposure with indicators of health-related harm, enabling a more clinically meaningful assessment of mobbing.

An innovative response to these challenges is offered by the SDM questionnaire (*Skala Doświadczania Mobbingu*), originally developed in Poland based on both theoretical models and empirical research, including clinical interviews with individuals exposed to prolonged workplace harassment [16,17]. The SDM is grounded in an interactional stress framework, in which exposure to negative acts is conceptualised as a chronic stressor that may lead to diverse cognitive, emotional, and psychosomatic responses. Importantly, this approach assumes that bare exposure to negative acts is not equivalent to the development of clinically significant victimisation by mobbing. The central innovation of the SDM lies in its operationalisation of health-related harm as a latent construct assessed through multiple indicators, rather than through a single self-labelling item [17,18]. This integrative approach enables a diagnostically meaningful distinction between exposure to mobbing and victimisation by mobbing and directly addresses key methodological limitations of existing instruments [14,15].

Following empirical refinement, shortened versions of the SDM were developed, including the 20-item version (SDM-20) as a screening tool and the 34-item version (SDM-34) for more detailed assessment. Both versions demonstrated strong psychometric properties in a large, representative Polish sample [18]. However, cross-cultural validation is necessary to establish the generalisability of this measurement approach. Preliminary evidence from a Polish–Spanish study using the original SDM suggests its relevance in Spanish organisational settings [20].

Against this background, the aim of the present study was to psychometrically validate the Spanish version of the SDM-20 as an occupational health screening instrument. Specifically, the authors examined whether the original 3-factor structure – integrating behavioural exposure and health-related harm – could be replicated in a Spanish working population and whether the adapted version would demonstrate satisfactory reliability and construct validity.

Based on the theoretical framework of the SDM and prior validation studies, the authors formulated the following hypotheses (H):

– H1: the 3-factor structure of the SDM-20 will be replicated in the Spanish sample,
– H2: the model will demonstrate good fit indices,
– H3: the subscales will show high internal consistency,
– H4: the latent factors will exhibit appropriate interrelations while remaining distinct,
– H5: the SDM-20 will demonstrate convergent validity with measures of workplace bullying and burnout, and divergent validity with organisational climate,
– H6: the instrument will demonstrate measurement invariance across gender.

## MATERIAL AND METHODS

### Participants

Data were collected online from 775 Spanish working adults in Andalusia using convenience sampling. After excluding 22 respondents (2.8% of the original sample) due to missing data, the final sample comprised 753 participants (65.5% women) aged 18–66 years (mean [M] ± standard deviation [SD] 39.45±12.75 years). Participants were employed in the public (N = 348, 46.2%) and private (N = 353, 46.9%) sectors, with 52 individuals (6.9%) not reporting sector affiliation. The sample represented a wide range of work experience: 166 participants (22.0%) had <1 year of experience, 176 (23.4%) had 1–3 years, 108 (14.3%) had 3–6 years, 84 (11.2%) had 6–10 years, and 219 (29.1%) had >10 years.

### Instruments

The primary instrument was the shortened SDM-20, designed for occupational health screening. The questionnaire comprises 2 behavioural exposure subscales: person-related mobbing (IDM_P – *Scale of Exposure to Person-Related Mobbing*, where IDM is derived from the Polish term *inwentarz działań mobbingowych* [inventory of mobbing behaviours] and P denotes “person-related”) (7 items, Cronbach's α = 0.91) and work-related mobbing (IDM_W – *Scale of Exposure to Work-Related Mobbing*, where W denotes “work-related*”*) (6 items, Cronbach's α = 0.86), as well as 1 cognitive-emotional subscale assessing perceived harm and victimisation (ODC – *Scale of Harm and Victimisation*, derived from the Polish term *odczuwane dolegliwości i cierpienie* [perceived harm and suffering]) (7 items, Cronbach's α = 0.92).” Items are rated on a 5-point Likert scale ranging from 1 (never) to 5 (always). The instrument's 3-factor model was previously validated in a large, nationally representative Polish sample (N = 2500). Confirmatory factor analysis (CFA) demonstrated satisfactory model fit (root mean square error of approximation (RMSEA) = 0.067, 90% confidence interval (CI): 0.063–0.071, standardized root mean square residual (SRMR) = 0.037, comparative fit index (CFI) = 0.941, Tucker–Lewis index (TLI) = 0.933 [18]).

The present study employed the Spanish version of the SDM-20 derived from the previously adapted 64-item SDM questionnaire. The original Spanish adaptation was developed within a collaborative Polish–Spanish research project in accordance with International Test Commission guidelines, including forward–backward translation, expert review, and evaluation of conceptual equivalence [20]. The 20-item screening version was obtained by retaining the items corresponding to the final factor structure established in the revised Polish SDM-20 [18]. As these items had already undergone linguistic and cultural adaptation, no further translation procedures were required. All items in Spanish and English are presented in Table 1. Furthermore, to support methodological transparency and facilitate the practical application of the instrument, the full Spanish version of the SDM-20 questionnaire is provided in Appendix 1.

**Table 1:** Content of the SDM-20 items in Spanish and English, with corresponding item codes from the original SDM-64 questionnaire – the item selection was based on the revised Polish SDM-20 structure established in a nationally representative sample (N = 2500) [18]

Item	SDM-64 code	Item content (Spanish)	Item content (English)	Subscale
1	SDM_14	Me ocultan información y datos importantes para llevar a cabo mi trabajo	Important data and information needed to complete tasks are concealed from me	IDM_W
2	SDM_22	Mi carrera está obstaculizada y retrasada	My career is impeded and delayed	IDM_W
3	SDM_24	Me hacen responsable de los errores de los demás	I am made to accept responsibility for the faults of others	IDM_W
4	SDM_29	Mi promoción profesional está impedida o bloqueada	My promotion is impeded or blocked	IDM_W
5	SDM_31	Recibo órdenes incoherentes y contradictorias	I receive inconsistent or contradictory orders	IDM_W
6	SDM_40	Soy excluido de asuntos importantes para la organización o la empresa	I am excluded from matters which are important to the organization	IDM_W
7	SDM_25	Algunos intentan burlarse de mí	Some people try to mock me	IDM_P
8	SDM_26	Algunas alusiones van dirigidas a mí, sin ser dichas abiertamente	Some allusions are directed at me without things being said openly	IDM_P
9	SDM_32	Soy objeto de miradas y gestos humillantes	I am the object of humiliating gestures and glances	IDM_P
10	SDM_33	Cada uno de mis errores es comentado y hecho público	My every mistake is publicized and commented upon	IDM_P
11	SDM_34	Evitan todo contacto conmigo, incluso el contacto visual	Any contact with me, including eye-contact, is avoided	IDM_P
12	SDM_36	Se difunden muchas mentiras sobre mí	A lot of gossip and lies are spread about me	IDM_P
13	SDM_38	Se ignoran o se ridiculizan mis opiniones y observaciones	My opinions and remarks are mocked or ignored	IDM_P
14	ODC_3	De camino al trabajo estoy nervioso/a y tengo ganas de volver a casa	On my way to work I am nervous, and I feel like going home	ODC
15	ODC_4	Después de salir del trabajo me siento mentalmente agotado/a y destrozado/a	After leaving the workplace I feel mentally exhausted and shattered	ODC
16	ODC_11	Cada vez me siento con menos confianza en el trabajo	I feel less and less confident at work	ODC
17	ODC_13	Los problemas en el trabajo me hacen perder la confianza en mí mismo/a	Problems at work make me stop believing in my worth	ODC
18	ODC_17	Estoy perdiendo confianza en mis habilidades, hasta el punto de preguntarme si éste es el camino correcto en mi trabajo	I am losing confidence in my abilities to the extent that I wonder whether this is the right line of work for me	ODC
19	ODC_19	Hay personas en el trabajo que tienen una influencia dañina sobre mi salud física y mental	Some people from my workplace have a damaging influence on both my mental and physical health	ODC
20	ODC_20	En presencia de algunas personas no puedo expresar lo que pienso libre y abiertamente	I can't speak my mind freely and calmly in the presence of some people at work	ODC

IDM_P – *Scale of Exposure to Person-Related Mobbing*; IDM_W – *Scale of Exposure to Work-Related Mobbing*; ODC – *Scale of Harm and Victimisation*; SDM – *Scale of Mobbing Experience* (*Skala Doświadczania Mobbingu*).

To examine convergent and divergent validity, 3 additional instruments were administered. Workplace bullying was assessed using the Spanish adaptation of the NAQ-R [19], a 23-item measure (including one item on sexual harassment) assessing exposure to negative acts during the previous 6 months on a 5-point Likert scale (1 – never to 5 – daily). The NAQ-R comprises 3 dimensions:

– personal bullying (e.g., “Being humiliated or ridiculed in connection with your work”),
– work-related bullying (e.g., “Someone withholding information which affects your performance”),
– physical bullying.

The scale has demonstrated excellent internal consistency and a stable 3-factor structure in large Spanish samples (α ranging 0.91–0.93).

Burnout was measured using the 9-item Spanish version of the *Maslach Burnout Inventory – General Survey* (MBI-GS9) [21,22]. The instrument comprises 3 subscales (3 items each):

– exhaustion (e.g., “I feel that my work is wearing me out”),
– cynicism (e.g., “I feel that in my work, I have become more insensitive and hard on people”),
– professional efficacy (reverse scored; e.g., “I believe that with my work I achieve something useful and valuable for others”).

Organizational climate was assessed using a 12-item version of the *First Organizational Climate/Culture Unified Search* (FOCUS-93) questionnaire [23]. In the present study, 4 subscales were employed:

– support (e.g., “Colleagues help each other to get the work done”),
– innovation (e.g., “New ideas are put into practice to improve work and its results”),
– goals (e.g., “The objectives to be achieved in a given period of time are clearly defined”),
– rules (e.g., “There is a strong emphasis on compliance with rules and procedures”).

### Procedure

The study protocol adhered to the Declaration of Helsinki and was approved by the Ethics Committee of the Institute of Psychology at the University of Wrocław, Poland. A cross-sectional online survey was conducted in January–April 2024. Participants were recruited via institutional mailing lists and professional and private social networks. Inclusion criteria were being currently employed, age ≥18 years, and residence in Spain

All participants provided informed consent prior to participation, in accordance with the General Data Protection Regulation (EU 2016/679), after receiving information on the study purpose, voluntary participation, the right to withdraw at any time, and data protection procedures.

Data were collected anonymously, with no directly identifying information obtained, and IP address collection disabled or removed prior to data export. Data were stored on a password-protected computer of the principal investigator, with appropriate technical safeguards (including encryption and restricted access), and access was limited to the research team.

Given the inclusion of mobbing-related items, participants were informed about the potentially sensitive nature of some questions. Participation was voluntary, and respondents could discontinue the survey at any time without penalty. The online format allowed completion in a private setting, and information on psychological support resources was provided after survey completion.

Average completion time was approx. 15 min, and no financial compensation was offered. The study protocol was preregistered on the Open Science Framework (OSF). Supplementary materials, R scripts, and the anonymized dataset are available on OSF [24].

### Data analysis

The preregistered analysis plan was followed. First, basic psychometric properties, including M, SD, medians (Me), ranges, interquartile ranges, and normality, were calculated for all items, scales, and subscales. Normality was assessed using kurtosis and skewness, adhering to recommended guidelines (kurtosis ≤|7|, skewness ≤|2|) [25]. Inter-item correlations were examined for dimensionality, and item-subscale correlations for discrimination properties. Internal reliability was assessed using Cronbach's α and McDonald's ω [26].

Next, CFA was conducted. Because the SDM-20 uses a 5-point response scale, the data were treated as ordinal, and the robust unweighted least squares estimator (ULSMV) was applied to the polychoric correlation matrix to ensure robust parameter estimation and accurate fit indices. The ULSMV estimator is particularly well suited for addressing non-normality in the data (specifically the substantial skewness and kurtosis values observed in the sample; see Results section for details). Model fit was evaluated using CFI, TLI, RMSEA, and SRMR, with commonly accepted cut-off criteria [27]. Latent factor correlations were examined to assess construct distinctiveness.

Subsequently, convergent and divergent validity were examined via correlations between SDM-20 scores and measures of workplace bullying, burnout, and organisational climate. Positive correlations were expected with workplace bullying and burnout, whereas negative correlations were expected with organisational climate indicators.

Finally, invariance across gender was assessed using multigroup CFA. Configural invariance was tested first, followed by metric (loadings constrained) and scalar (intercepts constrained) invariance. Models were compared using recommended criteria: a change in CFI and TLI ≤ 0.01, a change in RMSEA ≤ 0.015, and a change in SRMR ≤ 0.01, indicating no meaningful deterioration in model fit [28,29]. All statistical analyses were performed in R (v. 4.4.1) [30].

## RESULTS

### Descriptive statistics

Psychometric properties of all SDM-20 items and subscales, including means, standard deviations, medians, skewness, and kurtosis, are presented in Table 2 and 3, respectively. Notably, several items exhibited substantial positive skewness and kurtosis. This finding is theoretically consistent with the nature of workplace mobbing, as it is a low-prevalence but high-impact phenomenon in which a floor effect is common in general working populations. Inter-item correlations are reported in Table S1, and item–subscale correlations in Table S2 available in the Open Science Framework (OSF) repository [24].

**Table 2:** Descriptive statistics and distributional properties of the Spanish SDM-20 items in a sample of Spanish employees (N = 753), Spain

Item code^a^	M	SD	95% CI for M	Me	Min.	Max	Q1	Q3	Skewness	Kurtosis
SDM_14	1.833	1.098	1.754–1.911	1	1	5	1	2	1.275	3.816
SDM_22	1.892	1.173	1.809–1.976	1	1	5	1	3	1.204	3.499
SDM_24	1.834	1.055	1.759–1.909	1	1	5	1	2	1.254	3.902
SDM_29	1.870	1.213	1.783–1.957	1	1	5	1	2	1.323	3.678
SDM_31	2.130	1.157	2.047–2.213	2	1	5	1	3	0.868	2.927
SDM_40	1.919	1.153	1.836–2.001	2	1	5	1	2	1.226	3.665
SDM_25	1.441	0.849	1.380–1.502	1	1	5	1	2	2.171	7.417
SDM_26	1.734	1.015	1.662–1.807	1	1	5	1	2	1.488	4.664
SDM_32	1.320	0.727	1.268–1.372	1	1	5	1	1	2.663	10.524
SDM_33	1.567	0.907	1.502–1.632	1	1	5	1	2	1.827	6.135
SDM_34	1.260	0.665	1.213–1.308	1	1	5	1	1	3.119	13.732
SDM_36	1.448	0.860	1.386–1.509	1	1	5	1	2	2.315	8.401
SDM_38	1.501	0.877	1.438–1.563	1	1	5	1	2	2.064	7.268
ODC_3	1.776	1.066	1.699–1.852	1	1	5	1	2	1.470	4.535
ODC_4	2.616	1.183	2.532–2.701	2	1	5	2	3	0.448	2.378
ODC_11	1.821	1.065	1.745–1.897	1	1	5	1	2	1.419	4.476
ODC_13	1.907	1.131	1.826–1.988	2	1	5	1	2	1.282	3.858
ODC_17	1.817	1.098	1.738–1.895	1	1	5	1	2	1.356	4.031
ODC_19	1.876	1.108	1.797–1.956	2	1	5	1	2	1.254	3.811
ODC_20	2.193	1.223	2.105–2.280	2	1	5	1	3	0.772	2.591

Abbreviation as in Table 1.

a Original numeration from the SDM-64.

**Table 3:** Basic psychometric properties of the Spanish version of the SDM-20 in a sample of Spanish employees (N = 753), Spain

Scale	M	SD	95% CI	Me	Min.	Max	Q1	Q3	Cronbach's α	McDonald's ω	Skewness	Kurtosis
Means												
SDM-20	1.788	0.718	1.736–1.839	1.600	1.000	5.000	1.250	2.100	0.942	0.957	1.458	5.325
IDM work-related	1.913	0.887	1.850–1.976	1.667	1.000	5.000	1.167	2.333	0.868	0.901	1.228	4.226
IDM personal	1.467	0.684	1.418–1.516	1.143	1.000	5.000	1.000	1.714	0.909	0.936	2.157	8.100
IDM behavioral	1.673	0.713	1.622–1.724	1.462	1.000	5.000	1.154	1.923	0.921	0.944	1.655	5.970
ODC cognitive-emotional	2.001	0.884	1.938–2.064	1.714	1.000	5.000	1.286	2.429	0.896	0.931	1.172	3.972
Sums												
SDM-20	35.754	14.368	34.726–36.782	32.000	20.000	100.000	25.000	42.000	0.942	0.957	1.458	5.325
IDM work-related	11.478	5.321	11.097–11.859	10.000	6.000	30.000	7.000	14.000	0.868	0.901	1.228	4.226
IDM personal	10.271	4.787	9.928–10.613	8.000	7.000	35.000	7.000	12.000	0.909	0.936	2.157	8.100
IDM behavioral	21.749	9.273	21.086–22.412	19.000	13.000	65.000	15.000	25.000	0.921	0.944	1.655	5.970
ODC cognitive-emotional	14.005	6.189	13.563–14.448	12.000	7.000	35.000	9.000	17.000	0.896	0.931	1.172	3.972

Abbreviation as in Table 1.

### Factor structure and reliability

Confirmatory factor analysis showed that both the 2-factor and 3-factor models fit the data well (Table 4); however, the 3-factor solution demonstrated slightly superior fit indices (CFI = 0.990, TLI = 0.988, RMSEA = 0.054, SRMR = 0.049). Latent correlations among the 3 SDM-20 dimensions – IDM_W, IDM_P, and ODC – were substantial but remained below the commonly accepted threshold for construct redun dancy (r ≥ 0.80). Specifically, all correlations were >0.703 but <0.80, supporting the distinctiveness and uniqueness of the latent constructs in line with recommended criteria [31]. All subscales demonstrated very high internal reliability (Cronbach's α = 0.868, 0.909, 0.896 and ω = 0.901, 0.936, 0.931 respectively).

**Table 4:** A summary of the results of the confirmatory factor analysis (CFA) of the Spanish version of the SDM-20 in a sample of Spanish employees (N = 753), Spain

Factor	Estimate	SE	z	p	Standardized estimate
CFA – 2-factor structure					
IDM					
SDM_14	1				0.712
SDM_22	1.015	0.070	14.511	<0.001	0.676
SDM_24	0.963	0.058	16.667	<0.001	0.714
SDM_29	1.023	0.065	15.720	<0.001	0.659
SDM_31	1.075	0.060	17.984	<0.001	0.726
SDM_40	1.034	0.063	16.356	<0.001	0.701
SDM_25	0.779	0.060	12.903	<0.001	0.718
SDM_26	0.980	0.059	16.526	<0.001	0.754
SDM_32	0.630	0.061	10.30	<0.001	0.677
SDM_33	0.801	0.060	13.349	<0.001	0.690
SDM_34	0.499	0.059	8.508	<0.001	0.586
SDM_36	0.756	0.065	11.699	<0.001	0.687
SDM_38	0.820	0.055	14.981	<0.001	0.731
ODC					
ODC_3	1				0.665
ODC_4	1.194	0.081	14.695	<0.001	0.715
ODC_11	1.287	0.084	15.371	<0.001	0.856
ODC_13	1.173	0.077	15.23	<0.001	0.735
ODC_17	1.218	0.080	15.186	<0.001	0.786
ODC_19	1.171	0.089	13.108	<0.001	0.749
ODC_20	1.226	0.097	12.690	<0.001	0.710
IDM					
ODC	0.437	0.047	9.246	<0.001	0.789
CFA – 3-factor structure					
IDM					
personal					
SDM_25	1				0.794
SDM_26	1.256	0.078	16.135	<0.001	0.834
SDM_32	0.810	0.065	12.442	<0.001	0.750
SDM_33	1.025	0.079	12.986	<0.001	0.761
SDM_34	0.641	0.062	10.255	<0.001	0.649
SDM_36	0.973	0.061	15.942	<0.001	0.762
SDM_38	1.049	0.068	15.351	<0.001	0.806
work-related					
SDM_14	1				0.738
SDM_22	1.018	0.070	14.607	<0.001	0.704
SDM_24	0.960	0.058	16.647	<0.001	0.738
SDM_29	1.028	0.065	15.730	<0.001	0.687
SDM_31	1.079	0.060	18.014	<0.001	0.756
SDM_40	1.031	0.063	16.335	<0.001	0.725
ODC					
ODC_3	1				0.665
ODC_4	1.194	0.081	14.692	<0.001	0.715
ODC_11	1.287	0.084	15.366	<0.001	0.856
ODC_13	1.173	0.077	15.226	<0.001	0.735
ODC_17	1.218	0.080	15.184	<0.001	0.786
ODC_19	1.17	0.089	13.100	<0.001	0.748
ODC_20	1.226	0.097	12.687	<0.001	0.710
IDM					
work-related					
IDM (personal)	0.423	0.050	8.432	<0.001	0.775
ODC	0.336	0.047	7.178	<0.001	0.703
personal					
ODC	0.441	0.048	9.202	<0.001	0.768

SE – standard error; z – z statistic.

Other abbreviation as in Table 1.

### Construct validity

Consistent with expectations, strong positive correlations between the SDM-20 and the NAQ-R were found, with the highest coefficients observed for the total scores and theoretically corresponding behavioural subscales (r = 0.579–0.811). Lower, although still significant, associations emerged for the NAQ-R physical/sexual bullying subscale. Moderate-to-strong relationships were also identified with the MBI-GS9 (r = 0.378–0.732), further supporting alignment with theoretically related constructs. In contrast, negative associations between the SDM-20 subscales and organizational climate dimensions (r = −0.239–[−0.546]) provided evidence of divergent validity (all p < 0.001) (Table 5).

**Table 5:** Convergent and divergent validity of the Spanish version of the SDM-20 questionnaire

Scale and subscale	Pearson's r correlations																
	1	2	3	4	5	6	7	8	9	10	11	12	13	14	15	16	17
1. SDM-20 (total)	–																
2. IDM personal	0.860	–															
3. IDM work-related	0.889	0.682	–														
4. ODC cognitive – emotional	0.895	0.641	0.680	–													
5. NAQ-R (total)	0.811	0.769	0.706	0.684	–												
6. NAQ-R personal bullying	0.776	0.791	0.645	0.639	0.971	–											
7. NAQ-R work-related bullying	0.737	0.579	0.704	0.661	0.864	0.723	–										
8. NAQ-R physical/sexual bullying	0.385	0.438	0.320	0.281	0.579	0.576	0.351	–									
9. Burnout (MBI-GS9 total)	0.628	0.442	0.527	0.666	0.568	0.509	0.588	0.253	–								
10. Burnout – cynicism	0.476	0.378	0.421	0.451	0.480	0.435	0.485	0.225	0.775	–							
11. Burnout – emotional exhaustion	0.640	0.407	0.513	0.732	0.543	0.476	0.589	0.208	0.786	0.477	–						
12. Burnout – personal accomplishment	0.182	0.133	0.158	0.184	0.154	0.146	0.140	0.099	0.553	0.154	0.113	–					
13. Organizational climate (FOCUS total)	–0.530	–0.409	–0.501	–0.486	–0.421	–0.392	–0.410	–0.187	–0.458	–0.285	–0.406	–0.273	–				
14. Climate – support	–0.451	–0.407	–0.383	–0.405	–0.382	–0.377	–0.321	–0.199	–0.375	–0.262	–0.328	–0.197	0.816	–			
15. Climate – innovation	–0.546	–0.416	–0.522	–0.500	–0.438	–0.403	–0.433	–0.195	–0.445	–0.264	–0.403	–0.268	0.892	0.692	–		
16. Climate – goals	–0.453	–0.317	–0.451	–0.419	–0.337	–0.298	–0.365	–0.119	–0.408	–0.243	–0.375	–0.239	0.889	0.595	0.753	–	
17. Climate – rules	–0.334	–0.239	–0.327	–0.310	–0.263	–0.245	–0.256	–0.122	–0.314	–0.191	–0.258	–0.216	0.780	0.482	0.544	0.635	

B – bullying; FOCUS – *First Organizational Climate / Culture Unified Search* questionnaire (Spanish short version); MBI-GS9 – *Maslach Burnout Inventory – General Survey*, 9-item version; NAQ-R – *Negative Acts Questionnaire – Revised*. Other abbreviations as in Table 1.

All coefficients are Pearson's r correlation coefficients.

All correlations are significant at p < 0.001.

All external measures used to assess convergent and divergent validity demonstrated satisfactory reliability, with Cronbach's α values >0.78 and McDonald's ω values >0.80 (all p < 0.001). Detailed reliability coefficients are reported in Table S3, available in the OSF repository [24].

### Measurement invariance

Multigroup CFA indicated that the SDM-20 operates equivalently across men and women. The configural, metric, and scalar models all demonstrated good fit supporting the conclusion that the scale functions similarly across gender groups. Detailed results are presented in Table S4, available in the OSF repository [24].

## DISCUSSION

### Principal findings

The present study aimed to evaluate the psychometric properties of the Spanish version of the SDM-20 and to assess its suitability as a screening instrument for workplace mobbing within occupational health contexts. Overall, the findings provide robust evidence for the instrument's structural validity, internal consistency, and construct validity, directly addressing the methodological gap identified in existing assessment approaches [15,18], and supporting its application in occupational medicine and psychosocial risk assessment [2,12].

Confirmatory factor analysis replicated the original 3-factor structure, demonstrating very good fit to the data (supporting H1 and H2). This finding confirms that the distinction between person-related exposure, work-related exposure, and the latent construct of health-related harm is empirically stable in the Spanish context, supporting the clinical relevance of differentiating exposure from health-impairing victimisation [14,17]. All SDM-20 subscales met or exceeded recommended thresholds for internal reliability (confirming H3), in line with established standards for screening instruments used in clinical and occupational health settings [26].

Evidence for convergent validity was provided by strong associations between the SDM-20 behavioural exposure subscales and the NAQ-R, indicating that the instrument adequately captures exposure to mobbing. Moreover, negative associations with organisational climate dimensions provided evidence of divergent validity (fully supporting H5). Importantly, significant correlations with burnout dimensions and the moderate-to-high associations between behavioural exposure and the harm subscale support the theoretical assumption that exposure to negative behaviours and the experience of harm and victimisation, although related, represent distinct psychological realities (consistent with H4). This differentiation is central to understanding mobbing not merely as a pattern of behaviours, but as an interactional process with clinically relevant consequences [14–18]. Furthermore, the relatively weaker associations between SDM-20 and physical/sexual aggression are consistent with contemporary conceptualisations of workplace mobbing as predominantly involving subtle, non-physical forms of mistreatment, a pattern also reflected in prior empirical findings [6,17,18]. The reliance on correlation-based validity evidence in this study is consistent with established standards for instrument validation [32]. The observed pattern of convergent and divergent associations was theoretically expected and is consistent with findings from the original Polish validation [18], supporting the generalisability of the SDM-20 construct validity across socio-organisational settings. Finally, multigroup analyses confirmed that the scale functions equivalently for men and women (supporting H6).

### Theoretical and methodological implications

The successful validation of the SDM-20 supports the cross-cultural applicability of an interactional approach to measuring workplace mobbing. This approach directly addresses a central limitation of the dominant behavioural experience method (BEM), which conceptualises mobbing primarily in terms of the frequency of negative acts [6]. Although BEM-based instruments are well established and widely used, they do not directly operationalise psychological harm, despite harm being a defining element of mobbing and a prerequisite for assessing its clinical severity [14–16].

Previous attempts to bridge this gap by combining behavioural checklists with single self-labelling items have yielded only partial solutions [5]. Self-labelling approaches remain vulnerable to cognitive biases and individual differences in awareness, and they often fail to detect subclinical or emerging symptoms of victimisation. In contrast, the SDM-20 conceptualises harm as a latent construct derived from multiple cognitive, emotional, and psychosomatic indicators [14,17,18]. The present findings, in line with the Polish validation study, extend prior evidence by demonstrating that the proposed factorial structure retains its psychometric integrity and theoretical consistency in a distinct cultural and organisational setting [18].

By explicitly operationalising health-related harm, the SDM-20 aligns with contemporary stress–strain models in occupational health, in which workplace mobbing is conceptualised as a chronic psychosocial stressor with cumulative and potentially enduring effects on both mental and somatic functioning [7]. In this sense, the SDM-20 advances existing measurement frameworks by integrating behavioural exposure and health impairment within a single, diagnostically relevant model [17,18].

### Implications for occupational health practice

From a practical perspective, the validation of the SDM-20 has important implications for occupational medicine practitioners, psychologists, and organisational prevention services. First, the instrument facilitates differential diagnosis. By assessing both behavioural exposure and resulting harm, the SDM-20 enables practitioners to distinguish between employees who are exposed to negative acts (targets) and those who have internalised these experiences, resulting in psychosomatic impairment (victims). This distinction is critical for effective triage and intervention planning. Employees with high exposure but low harm scores may primarily benefit from organisational-level interventions, such as conflict resolution or mediation, whereas individuals with elevated harm scores require timely clinical assessment and support to prevent the development of chronic mental health conditions or prolonged work disability [2,10].

Second, the SDM-20 is particularly well suited for occupational health surveillance. Its brevity and strong psychometric properties make it an efficient tool for large-scale screening during periodic medical examinations. Early identification of the harm component supports secondary prevention by enabling the detection of employees at risk of health deterioration before the onset of long-term sickness absence [9,10]. Integrating such a measure into routine health monitoring systems shifts psychosocial risk management from descriptive prevalence estimation toward proactive protection of workers' mental health, in line with contemporary regulatory frameworks and occupational health surveillance requirements [33–35].

Beyond its clinical utility, the SDM-20 has important implications for occupational health practice in regulatory and medico-legal contexts [11,13]. By explicitly operationalising health-related harm through the ODC subscale, the instrument enables the documentation of clinically relevant impairment rather than mere exposure to negative acts. This feature is particularly relevant in the Spanish context, where technical guidelines for psychosocial risk management emphasise the need to demonstrate health-threatening effects of psychological violence at work [12]. In this sense, the SDM-20 facilitates the objectification of harm, supporting evidence-based decision-making in occupational health surveillance, fitness-for-work assessments, and compensation-related procedures [13].

Moreover, the brevity of the SDM-20 enhances its feasibility for large-scale implementation in routine occupational health examinations, including in small and medium-sized enterprises, where psychosocial risks are often under-assessed. By enabling early identification of health impairment related to mobbing exposure, the instrument supports secondary prevention efforts and may contribute to reducing long-term sickness absence and associated economic costs [9,12,35].

### Limitations and future directions

Several limitations should be acknowledged. First, the study was based on a convenience sample drawn from a single Spanish region, which may be affected by self-selection bias. Although the sample was heterogeneous in terms of age, sector, and work experience, it is not fully representative of the national workforce, which limits the generalisability of the findings. Future research should validate the SDM-20 in representative, stratified national samples and establish normative data and diagnostic cutoff scores for the Spanish population.

Second, the cross-sectional design precludes causal inferences regarding the temporal relationship between exposure to mobbing behaviours and the development of health-related harm. Longitudinal studies are needed to examine the dynamic interplay between exposure and symptom trajectories over time and to evaluate the predictive validity of the SDM-20 [10].

Third, the reliance on self-report measures introduces the possibility of common method bias. Future studies could strengthen the evidence base by incorporating objective indicators, such as sickness absence records or physiological markers of stress, although the collection of such data presents practical and ethical challenges in occupational settings [15,16].

Finally, while the present study validated the SDM-20 as a screening instrument, future research should explore the application of the more comprehensive SDM-34 version. The extended version may be particularly useful for in-depth clinical assessment in specialised occupational health units [13,18]. Despite these limitations, the present findings provide a strong empirical foundation for further validation work and support the use of the SDM-20 in applied occupational health settings.

## CONCLUSIONS

The present study successfully validated the Spanish version of the SDM-20, demonstrating a stable 3-factor structure, high internal consistency, and satisfactory convergent and divergent validity. These findings support the use of the SDM-20 as a reliable and efficient instrument for assessing workplace mobbing in Spanish-speaking occupational contexts.

The central conclusion of this research is that integrating behavioural indicators of exposure with a validated measure of psychological and health-related harm is not only methodologically feasible but also diagnostically necessary [14,16,18]. By enabling a clear distinction between exposure and victimisation, the SDM-20 overcomes the limitations of traditional frequency-based measures and provides occupational health professionals with a precise tool for early risk detection [15,18]. In doing so, the SDM-20 supports more effective prevention strategies and targeted interventions aimed at protecting employees' mental health and well-being, while also responding to broader public health and regulatory demands for evidence-based psychosocial risk management [33–35]. By enabling the objective assessment of health-related harm and facilitating early detection of mobbing-related impairment, the SDM-20 may contribute to reducing sickness absence, work disability, and the substantial societal and economic costs associated with workplace mobbing [9,10,36]. As such, the instrument provides a foundation for the development of diagnostically valid standards in occupational health practice and supports the over-arching public health goal of promoting safe, sustainable, and health-supportive work environments [2,33–35].

## Appendix 1. *Scale of Mobbing Experience (Skala Doświadczania Mobbingu* – SDM-20) questionnaire, Spanish version

A continuación se presentan una serie de situaciones que pueden ocurrir en el entorno laboral. Indique en qué medida dichas situaciones le han ocurrido en su trabajo actual. Para cada afirmación, evalúe la frecuencia con la que ha experimentado estas situaciones, utilizando la siguiente escala de respuesta:

1 – nunca | 2 – rara vez | 3 – a veces | 4 – a menudo | 5 – muy frecuentemente

1. Me ocultan información y datos importantes para llevar a cabo mi trabajo.
2. Mi carrera está obstaculizada y retrasada.
3. Me hacen responsable de los errores de los demás.
4. Mi promoción profesional está impedida o bloqueada.
5. Recibo órdenes incoherentes y contradictorias.
6. Soy excluido de asuntos importantes para la organización o la empresa.
7. Algunos intentan burlarse de mí.
8. Algunas alusiones van dirigidas a mí, sin ser dichas abiertamente.
9. Soy objeto de miradas y gestos humillantes.
10. Cada uno de mis errores es comentado y expuesto públicamente.
11. Evitan todo contacto conmigo, incluso el contacto visual.
12. Se difunden muchas mentiras sobre mí.
13. Se ignoran o se ridiculizan mis opiniones y observaciones.

A continuación, se presentan algunas reacciones que pueden aparecer como respuesta a conductas negativas y situaciones difíciles en el entorno laboral. Indique con qué frecuencia ha experimentado cada una de ellas en su trabajo actual, utilizando la siguiente escala de respuesta:

1 – nunca | 2 – rara vez | 3 – a veces | 4 – a menudo | 5 – muy frecuentemente

14. De camino al trabajo estoy nervioso/a y tengo ganas de volver a casa.
15. Después de salir del trabajo me siento mentalmente agotado/a y destrozado/a.
16. Cada vez me siento con menos confianza en el trabajo.
17. Los problemas en el trabajo me hacen perder la confianza en mí mismo/a.
18. Estoy perdiendo confianza en mis habilidades, hasta el punto de preguntarme si este es el camino correcto en mi trabajo.
19. Hay personas en el trabajo que tienen una influencia dañina sobre mi salud física y mental.
20. En presencia de algunas personas no puedo expresar libre y abiertamente lo que pienso.

**Answer key:** *Scale of Exposure to Work-Related Mobbing* (IDM_W) (6 items: 1–6); *Scale of Exposure to Person-Related Mobbing* (IDM_P) (7 items: 7–13); *Scale of Harm and Victimisation* (ODC) (7 items: 14–20).
